# Simvastatin Suppresses Airway IL-17 and Upregulates IL-10 in Patients With Stable COPD

**DOI:** 10.1378/chest.14-3138

**Published:** 2015-06-04

**Authors:** Kittipong Maneechotesuwan, Adisak Wongkajornsilp, Ian M. Adcock, Peter J. Barnes

**Affiliations:** From the Division of Respiratory Diseases and Tuberculosis (Dr Maneechotesuwan), Department of Medicine, and Department of Pharmacology (Dr Wongkajornsilp), Faculty of Medicine Siriraj Hospital, Mahidol University, Bangkok, Thailand; and Airway Section (Drs Adcock and Barnes), National Heart and Lung Institute, Imperial College, London, England.

## Abstract

**BACKGROUND:**

Statins have immunomodulatory properties that may provide beneficial effects in the treatment of COPD. We investigated whether a statin improves the IL-17/IL-10 imbalance in patients with COPD, as has previously been demonstrated in patients with asthma.

**METHODS:**

Thirty patients with stable COPD were recruited to a double-blind, randomized, controlled, crossover trial comparing the effect of simvastatin, 20 mg po daily, with that of a matched placebo on sputum inflammatory markers and airway inflammation. Each treatment was administered for 4 weeks separated by a 4-week washout period. The primary outcome was the presence of T-helper 17 cytokines and indoleamine 2,3-dioxygenase (IDO) in induced sputum. Secondary outcomes included sputum inflammatory cells, FEV_1_, and symptoms using the COPD Assessment Test (CAT).

**RESULTS:**

At 4 weeks, there was a significant reduction in sputum IL-17A, IL-22, IL-6, and CXCL8 concentrations (mean difference, −16.4 pg/mL, *P* = .01; −48.6 pg/mL, *P* < .001; −45.3 pg/mL, *P* = .002; and −190.9 pg/mL, *P* = .007, respectively), whereas IL-10 concentrations, IDO messenger RNA expression (fold change), and IDO activity (kynurenine to tryptophan ratio) were markedly increased during simvastatin treatment compared with placebo treatment periods (mean difference, 24.7 pg/mL, *P* < .001; 1.02, *P* < .001; and 0.47, *P* < .001, respectively). The absolute sputum macrophage count, proportion of macrophages, and CAT score were reduced after simvastatin compared with placebo (mean difference, −0.16 × 10^6^, *P* = .004; −14.1%, *P* < .001; and −3.2, *P* = .02, respectively). Values for other clinical outcomes were similar between the simvastatin and placebo treatments.

**CONCLUSIONS:**

Simvastatin reversed the IL-17A/IL-10 imbalance in the airways and reduced sputum macrophage but not neutrophil counts in patients with COPD.

**TRIAL REGISTRY:**

ClinicalTrials.gov; No.: NCT01944176; www.clinicaltrials.gov

Cytokines play a critical role in the pathobiologic processes of COPD, including altered innate immune response, chronic inflammation, emphysema, and small airway fibrosis.^1^ Proinflammatory cytokines of potential importance include tumor necrosis factor-α, interferon-γ, IL-1β, IL-17, and IL-6. COPD has been associated with an increased IL-17 response directed against innocuous antigens.^2^ IL-17 promotes chronic airway inflammation by primarily acting on the lung epithelium through the upregulation of proinflammatory cytokines and chemokines.^3,4^ Genetic deletion of IL-17A attenuated cigarette smoke-induced inflammation and alveolar type 2 apoptosis in mice.^5^ The expression of IL-17A in human bronchial submucosa was significantly increased in patients with COPD compared with healthy control subjects and normal smokers.^6,7^

Accumulating evidence suggests that CD4^+^ T cells, including T regulatory (Treg) cells and T-helper (Th)17 cells, possess a greater degree of plasticity in their differentiation options than previously appreciated.^8^ It appears that expression of Foxp3 by Treg cells or RORγt by Th17 cells may not be stable.^8^ Th17 cells produce IL-17 and IL-22, thereby boosting inflammation, while Treg cells express IL-10 and tumor growth factor-β, suppressing inflammation at least in part through the immunosuppressive effects of indoleamine 2,3-dioxygenase (IDO). The induction of IDO-enhanced tryptophan (Tryp) catabolism into kynurenine (Kyn), which can inhibit the accumulation of Th1 and Th17 cells at the site of inflammation and, therefore, attenuate the degree of inflammation.^9^ This may explain the imbalance between Th17 and Treg cytokines in COPD that we have previously documented.^10^ We have shown that sputum IL-17A concentrations are associated with COPD severity and inversely correlated with IL-10 concentrations, with reduced expression of the immunosuppressive enzyme IDO and its bioactivity.^11^ This may contribute, in part, to a further enhancement of airway inflammation in COPD.

Statins are 3-hydroxy-3-methyl-glutarylcoenzyme A reductase inhibitors that have been clinically used as lipid-lowering agents. Statins, however, have additional pleiotropic pharmacologic effects, including antiinflammatory, antioxidant, and immunomodulatory activities in vitro and in vivo.^12‐15^ The immunomodulatory effects of statins on Th17 cell- and IL-17-mediated inflammatory responses have been well established in autoimmune diseases, including multiple sclerosis in humans and experimental autoimmune encephalomyelitis in mice. Statins mediate their action via suppression of Th17 differentiation with the concurrent induction of Treg differentiation.^16^ Emerging evidence suggests that statins inhibit the release of airway neutrophilic mediators (CXCL8, IL-6, and granulocyte-macrophage colony-stimulating factor) from bronchial epithelial cells and suppress their upregulation by IL-17.^17^ Interestingly, all of these additional actions may counteract the neutrophilic inflammation-promoting effects of IL-17 in COPD. To date, there has been a paucity of studies exploring the antiinflammatory effects of statins in COPD and particularly on the reversal of IL-17A/IL-10 or Th17/Treg imbalance.

We conducted a double-blind, placebo-controlled crossover study to ascertain the effect of simvastatin on Th17 cytokines and Th17-polarizing cytokine expression and chronic airway inflammation in COPD. To our knowledge, this study is the first to show that simvastatin inhibits IL-17, IL-22, CXCL8, and IL-6, but enhances IL-10, IDO messenger RNA expression, and IDO biologic activity. However, these effects of simvastatin were not associated with the attenuation of airway neutrophilia but unexpectedly resulted in a marked decrease in macrophage numbers.

## Materials and Methods

### Study Design and Assignment

The study was a 4-week, randomized, double-blind, crossover study comparing the effect of oral simvastatin treatment (20 mg daily) with that of a matched placebo on sputum cytokine biomarkers and airway inflammation in COPD. After a 2-week run-in period, each treatment was administered to randomized patients for 4 weeks, separated by a 4-week washout period. Researchers and participants were blinded to allocation and had no access to the randomization code held by the data center until completion of the study. The conduct of this study complied with the Declaration of Helsinki. The study was approved by the Siriraj Institutional Review Board (Si323/2013). Written informed consent was obtained from each study patient prior to entry into the trial. The study was listed on all appropriate clinical trial registries.

During the 2-week run-in period and throughout the study, subjects continued their usual COPD medication and withdrew statin therapy for 4 weeks prior to the study entry if they were taking regular treatment. Further visits were undertaken at randomization and after 4 weeks (phase 1). After a washout period of 4 weeks, phase 2 of the crossover was started with a visit after 4 weeks. At each visit, spirometry was performed. Before and after each treatment period, sputum induction was performed and blood samples were taken to measure lipid levels and liver function. Tablets were counted at the end of each treatment period as a measure of treatment adherence.

### Patients

Patients with COPD aged 45 to 80 years were recruited from the COPD Outpatients Clinic at Siriraj Hospital. Patients with COPD met the following inclusion criteria: current or ex-smokers with a ≥ 10 pack-year history; postbronchodilator FEV_1_ to FVC ratio < 70% and FEV_1_ < 80% and ≥ 50% of predicted normal values (GOLD [Global Initiative for Chronic Obstructive Lung Disease] grade 2) at visit 1 (screening); and clinically stable as defined by no exacerbations in the previous 6 weeks.

Key exclusion criteria were a primary diagnosis of asthma; history of significant diseases other than COPD, including TB, lung cancer, and HIV; acute worsening of COPD that required treatment within 6 weeks prior to screening or between the screening and baseline visits; cognitive impairment; recent cardiovascular and cerebrovascular diseases within 6 months prior to study entry; current or previous use of immunosuppressive agents; and current administration of macrolides, azole antifungal drugs, and amlodipine.

Recruited study participants who were taking certain COPD medications at screening (ie, tiotropium, long-acting β_2_-agonists [LABAs], combination corticosteroid and LABA products, theophylline, oral β_2_-agonists) were permitted to continue these medications throughout the trial to maintain their clinical stability, with the exception of the study visit. Study participants were required to withhold all COPD medications (including inhaled corticosteroids [ICSs] and short-acting bronchodilators) for at least 8 h before the baseline visit and all subsequent study treatment visits.

At visit 2, study participants were randomized to one of two possible treatment sequences. Each sequence included either 4-week administration of simvastatin (20 mg/d) or placebo in a double-blind manner. Patients returned to the clinic for washout at visit 3, for treatment at visit 4, and for a final visit at visit 5. There was a washout interval between treatments of 4 weeks.

### Measurements

Demographic measurements were recorded on the first clinical visit (visit 1 screening). Induced sputum and blood samples were collected before and after treatment periods for analysis of sputum cytokines and differential leukocyte count and assessment of lipid profiles and high-sensitivity C-reactive protein (hsCRP). Spirometry, COPD Assessment Test (CAT) score, and impulse oscillometry were measured at all study visits. The primary end point was IL-17A, IL-10, and IL-6 levels, and IDO activity. Secondary end points included CAT scores, sputum neutrophil count, and postbronchodilator FEV_1_.

### Sputum Induction and Processing

Sputum induction was performed as previously described.^11^ The supernatants were kept frozen at −70°C until further analysis. For immunocytochemistry, cytospins were fixed with 4% paraformaldehyde (BDH Ltd) and stored at −20°C. Total cell counts were recorded with a hemocytometer, using Kimura staining. Cell viability was determined by Trypan blue exclusion before cytospins were undertaken. The slides were stained with May-Grunwald-Giemsa stain and differential cell counts were made by a blinded observer. A total of 400 inflammatory cells were counted on two slides for each sample in a blinded manner. Differential cell counts are expressed as the percentages of total inflammatory cells. Samples with cell viability > 70% and < 30% squamous cell contamination were considered adequate for analysis.

### Cytokine Assays

For detection of human IL-6, CXCL-8, IL-10, IL-17A, IL-22, and IL-23, sandwich enzyme-linked immunosorbent assays (ELISAs) were performed according to the manufacturer’s instructions (Abcam plc). The sensitivity was 0.8 pg/mL for IL-6, 1 pg/mL for CXCL8, 1.3 pg/mL for IL-10, 3 pg/mL for IL-17A, 5 pg/mL for IL-22, and < 20 pg/mL for IL-23. Cytokine output was normalized to the concentration of protein. It should be noted that the standard range of the ELISA assay used in this study was lower than that of the ELISA assays used by previous studies, possibly resulting in the baseline levels of some cytokines reported in this study being different from those reported in other studies. In addition, the differences of cytokine measurements in our study from others might be explained by the nature of our recruits. The majority of our subjects were treated with ICSs, whereas other studies recruited steroid-naive patients with COPD, those with mild COPD without treatment, or minority of patients with steroid-treated severe COPD.^18,19^ The difference in the nature of steroid use might contribute to the low basal levels of several cytokines.

### RNA Isolation and Real-Time Reverse Transcription-Polymerase Chain Reaction Analysis

Total RNA was extracted from whole sputum cell pellets by using an RNeasy Mini kit (Qiagen NV) according to the manufacturer’s instructions. Total RNA was reverse transcribed into cDNA by using Improm-II Reverse Transcription system (Promega Corporation). Real-time reverse transcription-polymerase chain reaction was performed with FastStart Universal SYBR Green Master (Roche Holding AG) with an ABI PRISM 7900 thermal cycler (Thermo Fisher Scientific Inc) according to the manufacturer’s instructions. The following IDO primers were purchased from First BASE Laboratory (BASE Life Sciences Holdings): sense: AGT CCG TGA GTT TGT CCT TTC AA, and antisense: TTT CAC ACA GGC GTC ATA AGC T; and glyceraldehyde-3-phosphate dehydrogenase sense: GAA ATC CCA TCA CCA TCT TCC, and antisense: AAA TGA GCC CCA GCC TTC TC. The 7300 System SDS software version 1.4.0.25 (Thermo Fisher Scientific Inc) was used to analyze the relative quantity of the target cDNA, according to the ΔΔCt method.

### Liquid Chromatography-Mass Spectrometry Analysis of L-Tryp and L-Kyn

Analysis of L-Tryp and L-Kyn was performed using a validated high-performance liquid chromatography with tandem mass spectrometry method as previously described.^20^ Briefly, 3-nitro-l-tyrosine was added to each sample as an internal standard, and protein precipitation was performed using 10% trichloroacetic acid. Chromatographic separation of the subsequent organic layer was carried out on liquid chromatography-tandem mass spectrometry with C18, 2.5 mm (50 × 3.0 mm internal diameter). A mobile phase consisting of acetonitrile and 0.1% formic acid (gradient condition) was delivered at a flow rate of 0.2 mL/min. Mass spectra were obtained using a Quattro Premier XE mass spectrometer (Micromass; Waters Corporation) operated in multiple reaction monitoring mode. Sample introduction and ionization were performed by electrospray ionization in the positive ion. The mass transition ion pair for L-Tryp protonated species ([M+H]+) and L-Kyn L-Tryp [M+H]+ ions was selected as mass to charge ratio 205.08 > 188.00 and 205.08 > 146.08, respectively. The mass transition ion-pair for 3-nitro-l-tyrosine [M+H]+ ions was selected as mass to charge ratio 227.02 > 181.03. The data acquisition was ascertained by Masslynx 4.1 software (Waters Corporation). The lower limit of detection for L-Tryp and L-Kyn in sputum supernatants were 0.05 μg/mL and 0.15 μg/mL, respectively. The best linear fit was achieved with a 1/x weighting factor, showing a mean correlation coefficient ≥ 0.998.

### Statistical Analyses

Baseline characteristics were described by number and percentage of patients for categorical variables and mean (±SD) for continuous variables. Response to simvastatin on lung function, induced sputum, and mediator levels vs placebo was assessed by general linear model for the standard 2 × 2 crossover design. When variables were unsuitable for this, the within-patient treatment differences were calculated and then analyzed by a paired *t* test for parametric and Wilcoxon signed rank test for nonparametric data. The effects of ICS treatment and smoking status on the response to simvastatin were analyzed by an unpaired *t* test. Significance at a level of 5% was accepted for the primary end point.

A sample size of 21 was calculated to have 80% power to detect a difference in means of 0.23 in IDO activity (the ratio of Kyn to Tryp) and 15 pg/mL in sputum IL-17A (primary end point), assuming an SD of difference of 0.35 and 20 pg/mL using a paired *t* test with a 5% two-sided significance level. A total of 30 patients were recruited to ensure that 21 patients completed the study. All data were analyzed using PASW statistics 18 (SPSS; IBM Corporation).

## Results

### Recruitment and Baseline Characteristics

**Figure 1 –:**
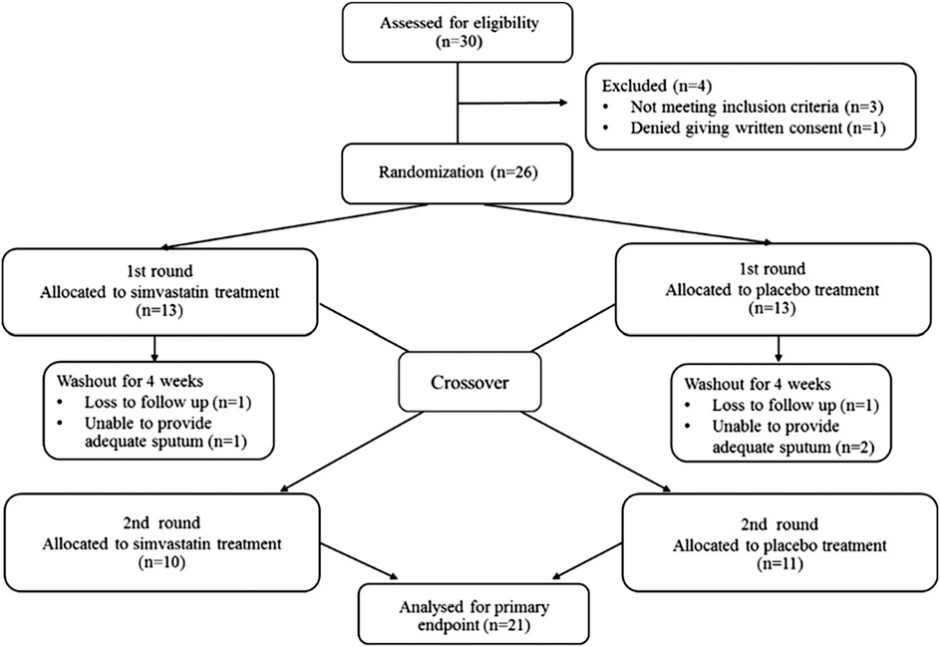
Flow of subjects through the study.

Screening visits were arranged for 30 patients; 26 patients were randomized to therapy because four were excluded prior to randomization (Fig 1). Of the 26 patients, 21 completed the treatment phase and five discontinued the study before completion, as indicated in Figure 1. Baseline demographic and clinical characteristics of the patients are shown in Table 1 and baseline inflammatory marker levels in Table 2. Almost all patients had symptomatic GOLD B COPD, according to GOLD classification. Distributions of baseline characteristics were similar for patients starting with placebo and those starting with simvastatin.

**TABLE 1 ]:** Demographic Data and Clinical Characteristics of Study Subjects

Clinical Parameters	All Patients (N = 21)
Age, y	68.4 ± 7.8
Smoking pack-y	36.7 ± 28.9
Ex-smoker to current smoker ratio, No.	16:5
Pre-BD FEV_1_/FVC, %	53.9 ± 11.5
Post-BD FEV_1_/FVC, %	55.7 ± 11.6
Post-BD FEV_1_, L	1.55 ± 0.6
Post-BD FVC, L	2.7 ± 0.7
ΔFEV_1_, mL	115.2 ± 108.0
BD reversibility, %	9.5 ± 8.9
Dlco, mL/mm Hg/min	57.4 ± 21.2
Dlco/Va, mL/mm Hg/min/L	62.4 ± 14.2
ICS/LABA use, %	38.1
LAMA use, %	4.7
Triple therapy, %	33.3
BD po, %	38.1
Previous statin therapy, %	38.1

Data are presented as mean ± SD unless otherwise indicated. BD = bronchodilator; Δ = change in; Dlco = diffusing capacity of the lung for carbon monoxide; ICS = inhaled corticosteroid; LABA = long-acting β_2_ agonist; LAMA = long-acting muscarinic antagonist; Va = alveolar volume.

**TABLE 2 ]:** Baseline and Before and After Each Treatment Induced-Sputum Cytology and Inflammatory Marker Levels

Variable	Mean ± SD	Placebo		*P* Value	Simvastatin		*P* Value
		Before	After		Before	After	
Induced-sputum cell counts and proportion (%)							
Total cells, × 10^6^	1.2 ± 0.5	0.88 (0.69-1.1)	0.94 (0.8-1.1)	.25	0.88 (0.75-1.1)	0.86 (0.64-1.2)	.67
Neutrophils, × 10^6^	0.8 ± 0.4	0.68 (0.5-0.8)	0.72 (0.57-0.9)	.13	0.62 (0.44-0.8)	0.59 (0.48-0.97)	.36
Neutrophils, %	67.5 ± 11.9	74.1 (69.1-85.2)	75.3 (68.6-86.7)	.21	70.0 (62.7-76.9)	77.7 (55.0-84.7)	.016
Eosinophils, × 10^6^	0.04 ± 0.1	0.001 (0.0-0.007)	0.003 (0.0-0.01)	.58	0.004 (0.0-0.01)	0.006 (0.0-0.03)	.29
Eosinophils, %	4.3 ± 12.3	0.2 (0.0-0.8)	0.0 (0.0-1.4)	.95	0.45 (0.0-1.6)	0.45 (0.0-2.5)	.68
Macrophage, × 10^6^	0.3 ± 0.1	0.14 (0.1-0.3)	0.16 (0.1-0.2)	.38	0.25 (0.17-0.29)	0.09 (0.05-0.17)	<.001
Macrophages, %	26.5 ± 9.4	19.7 (12.5-27.2)	18.1 (11.5-26.1)	.44	26.3 (20.7-32.8)	10.0 (7.2-16.9)	<.001
Lymphocytes, × 10^6^	0.0005 ± 0.002	0.0 (0.0-0.0)	0.0 (0.0-0.0)	.25	0.0 (0.0-0.0)	0.0 (0.0-0.002)	.2
Lymphocytes, %	0.02 ± 0.1	0.0 (0.0-0.0)	0.0 (0.0-0.0)	.25	0.0 (0.0-0.0)	0.0 (0.0-0.24)	.19
Airway epithelial cells, × 10^6^	0.02 ± 0.01	0.01 (0.005-0.02)	0.01 (0.006-0.02)	.97	0.02 (0.004-0.02)	0.03 (0.005-0.12)	.03
Airway epithelial cells, %	1.7 ± 1.6	1.6 (0.48-2.68)	0.96 (0.57-1.75)	.49	1.9 (0.36-2.64)	3.2 (0.57-16.5)	.019
Sputum mediators							
IL-17A, pg/mL	27.1 ± 17.8	20.2 (11.6-48.3)	22.5 (8.0-39.8)	.48	32.3 (14.5-45.5)	9.1 (5.4-21.2)	.0001
IL-22, pg/mL	63.2 ± 13.9	58.3 (51.8-63.4)	61.2 (49.6-63.4)	.36	69.9 (61.9-76.4)	25.0 (17.7-44.5)	< .001
IL-10, pg/mL	7.9 ± 1.9	8.1 (6.6-9.5)	8.3 (6.5-10.0)	.78	7.2 (5.9-9.0)	28.8 (17.5-41.7)	< .001
IL-6, pg/mL	41.5 ± 28.6	24.4 (10.7-52.3)	54.0 (16.2-79.5)	.02	37.2 (18.8-68.3)	29.6 (9.3-34.2)	< .001
CXCL8, pg/mL	624.1 ± 275.1	602.9 ± 239.9	627.0 ± 225.7	.7	612.7 ± 262.4	447.3 ± 205.1	< .001
IDO mRNA, fold change	1.0 (0.93-1.0)	0.99 (0.9-1.0)	1.8 (1.6-1.9)	< .001	1.0 (0.9-1.0)	2.7 (2.5-3.2)	< .001
IDO activity, Kyn/Tryp	0.88 ± 0.4	0.9 ± 0.49	0.7 ± 0.35	.03	0.77 ± 0.34	1.07 ± 0.31	.0002
Serum biomarker							
hs-CRP, mg/L	7.2 ± 16.0	6.0 ± 15.5	9.1 ± 25.3	1.0	7.3 ± 16.1	3.2 ± 6.2	.1

Data are given as mean ± SD or median (IQR). hsCRP = high-sensitivity C-reactive protein; IDO = indoleamine 2,3-dioxygenase; IQR = interquartile range; Kyn = kynurenine; mRNA = messenger RNA; Tryp = tryptophan.

### Changes in Inflammatory Biomarkers

#### IL-17 and IL-22:

**Figure 2 –:**
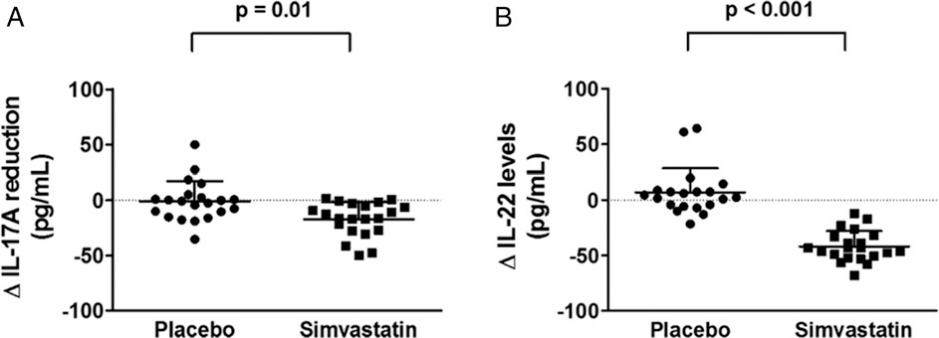
*A, B, The lowering of sputum IL-17A (A) and IL-22 (B) concentrations in response to simvastatin treatment to a greater extent than placebo. Data are expressed as mean (SD)*. Δ = *change in.*

To determine the effect of simvastatin on Th17 signature cytokine response in the airways of patients with COPD, IL-17A and IL-22 were quantified on the protein level in sputum supernatants of simvastatin- and placebo-treated patients with COPD. Patients with COPD who were treated with simvastatin showed a significant decrease of IL-17A levels compared with the baseline levels prior to treatment, whereas there was no significant reduction in control subjects treated with placebo (median [interquartile range (IQR)], 9.1 pg/mL [5.4-21.2 pg/mL] vs 32.3 pg/mL [14.5-45.5 pg/mL], *P* = .0001; 22.5 pg/mL [8.0-39.8 pg/mL] vs 20.2 pg/mL [11.6-48.3 pg/mL], *P* = .48, respectively). In addition, the magnitude of the reduction in sputum IL-17A concentrations was significant in patients with COPD treated with simvastatin compared with control subjects receiving placebo (mean difference, −16.4 pg/mL; 95% CI, −28.3 to −4.4; *P* = .01) (Fig 2A, Table 3). This suppression was not dependent upon the sequence of treatments either with simvastatin first and placebo second or vice versa (*P* = .74) (Table 3). There was no significant differences in the IL-17A response to simvastatin in patients taking ICSs compared with those not taking ICSs and between ex-smokers and current smokers (*P* = .9 and *P* = .5, respectively). Similarly, the magnitude of the reduction in sputum IL-22 levels was significant in patients with COPD treated with simvastatin compared with control subjects receiving placebo (mean difference, −48.6 pg/mL; 95% CI, −58.4 to −38.9; *P* < .001) (Fig 2B, Table 3). IL-22 response to simvastatin treatment was not affected by ICS treatment and smoking status (*P* = .09 and *P* = .4, respectively). However, IL-23 was undetectable in sputum (data not shown).

**TABLE 3 ]:** Treatment Differences in Sputum Cytology and Cytokines After 4 Wk of Treatment With Simvastatin Compared With Placebo

Variables	Placebo	Simvastatin	*P* Value	Treatment Difference, Simvastatin − Placebo (95% CI)	Effects by Order *P* Value
Δ Total cells, × 10^6^	0.01 ± 0.4	−0.09 ± 0.4	.55	−0.1 (−0.4 to 0.2)	.43
Δ Neutrophil, × 10^6^	0.03 ± 0.4	0.02 ± 0.3	.86	−0.02 (−0.24 to 0.2)	.38
Δ Neutrophils, %	2.2 ± 8.7	5.8 ± 16.9	.35	3.6 (−4.3 to 11.5)	.55
Δ Eosinophil, × 10^6^	−0.005 ± 0.04	0.01 ± 0.09	.44	0.01 (−0.02 to 0.05)	.85
Δ Eosinophils, %	−0.85 ± 2.7	1.3 ± 7.3	.17	2.2 (−1.0 to 5.4)	.65
Δ Macrophage, × 10^6^	−0.02 ± 0.1	−0.15 ± 0.1	.004	−0.16 (−0.26 to −0.06)	.3
Δ Macrophages, %	−0.9 ± 8.6	−15.2 ± 15.1	< .001	−14.1 (−21.0 to −7.1)	.49
Δ Lymphocyte, × 10^6^	0.001 ± 0.0	0.004 ± 0.0	.22	0.003 (−0.002 to 0.01)	.08
Δ Lymphocytes, %	0.06 ± 0.1	0.3 ± 0.7	.16	0.25 (−0.1 to 0.61)	.11
Δ Airway epithelial cell, × 10^6^	0.0 ± 0.04	0.08 ± 0.2	.08	0.08 (−0.01 to 0.18)	.21
Δ Airway epithelial cells, %	−0.38 ± 2.2	7.2 ± 11.4	.007	7.6 (2.3 to 12.9)	.08
Δ Sputum IL-17A, pg/mL	−0.89 ± 18.1	−17.2 ± 15.4	.01	−16.4 (−28.3 to −4.4)	.74
Δ Sputum IL-22, pg/mL	6.9 ± 21.9	−42.0 ± 13.9	< .001	−48.6 (−58.4 to −38.9)	.87
Δ Sputum IL-10, pg/mL	0.56 ± 2.8	25.4 ± 19.0	< .001	24.7 (15.9 to 33.6)	.65
Δ Sputum IL-6, pg/mL	23.7 ± 40.7	−21.6 ± 25.1	.002	−45.3 (−71.3 to −19.4)	.77
Δ Sputum CXCL8, pg/mL	24.1 ± 296.9	−165.4 ± 152.0	.007	−190.9 (−324.1 to −57.7)	.87
Δ IDO mRNA, fold change	0.97 ± 0.4	2.0 ± 0.8	< .001	1.02 (0.75 to 1.3)	.6
Δ IDO activity, Kyn/Tryp	−0.17 ± 0.3	0.29 ± 0.3	< .001	0.47 (0.28 to 0.65)	.34

Data are presented as mean ± SD. See Table 1 and 2 legends for expansion of abbreviations.

#### IL-10 and IDO:

**Figure 3 –:**
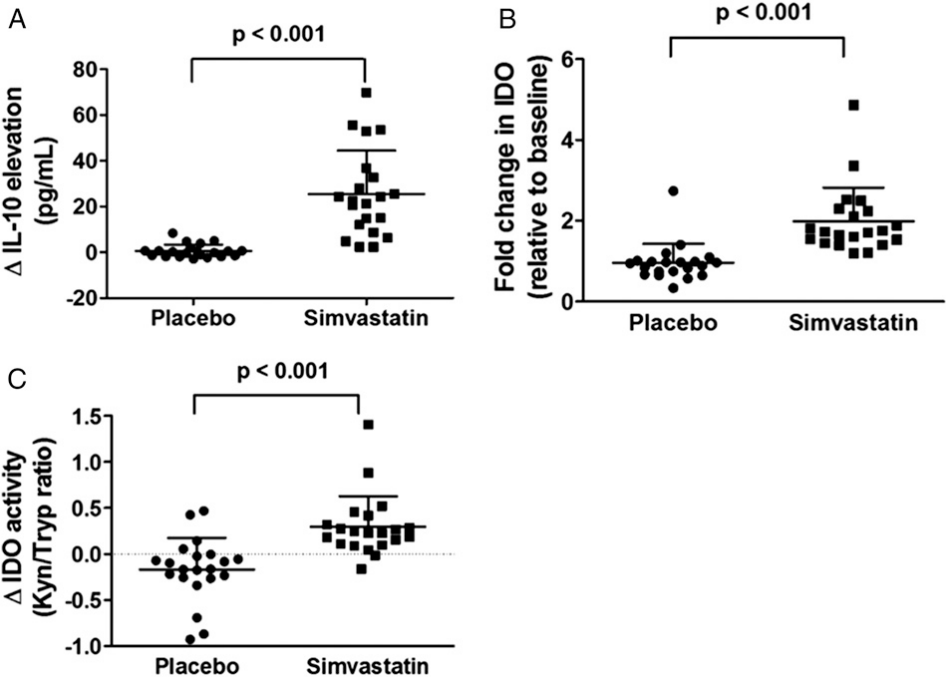
A, The elevation of sputum IL-10 concentrations was greater in response to simvastatin treatment than placebo. B, The same pattern was seen in IDO messenger RNA concentration. C, IDO activity also was elevated in response to simvastatin treatment to a greater extent than placebo. The data are expressed as mean (SD). IDO = indoleamine 2,3-dioxygenase; Kyn = kynurenine; Tryp = tryptophan. See Figure 2 legend for expansion of other abbreviation.

Patients with COPD treated with simvastatin had higher concentrations of IL-10 in sputum supernatants compared with the baseline levels, whereas there was no significant increase of IL-10 in patients with COPD who received placebo (median [IQR], 28.8 pg/mL [17.5-41.7 pg/mL] vs 7.2 pg/mL [5.9-9.0 pg/mL], *P* < .001; 8.3 pg/mL [6.5-10.0 pg/mL] vs 8.1 pg/mL [6.6-9.5 pg/mL], *P* = .78, respectively). In addition, the magnitude of the increase in IL-10 levels was significant in patients with COPD treated with simvastatin compared with subjects treated with placebo (mean difference, 24.7 pg/mL; 95% CI, 15.9-33.6; *P* < .001) (Fig 3A, Table 3). This effect was not dependent upon the sequence of treatments between simvastatin and placebo (*P* = .65). Patients without ICS treatment had a significantly greater response of IL-10 to simvastatin than those with ICS treatment, whereas smoking status did not affect IL-10 response (*P* = .037 and *P* = .9, respectively). IL-10 is an enhancer of IDO expression. We demonstrated an increase in IDO messenger RNA expression with its enzymatic activity with simvastatin treatment (mean difference in fold change, 1.02 [95% CI, 0.75-1.3], *P* < .001; 0.47 [95% CI, 0.28-0.65], *P* < .001, respectively) (Figs 3B, 3C, Table 3).

#### IL-6 and CXCL8:

**Figure 4 –:**
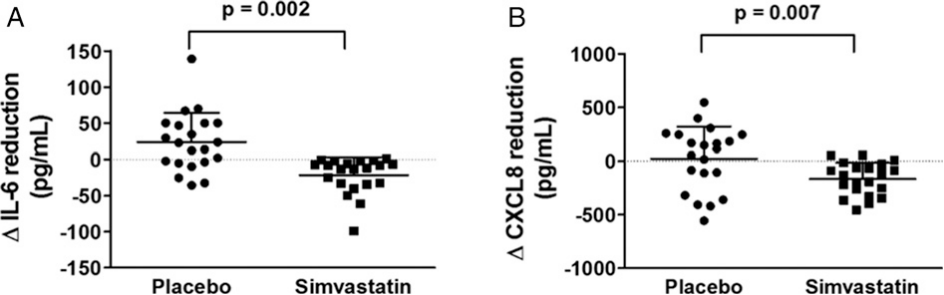
A, Sputum IL-6 concentration was lower in response to simvastatin treatment to a greater extent than placebo. B, The same pattern was seen in CXCL8 concentrations. The data are expressed as mean (SD). See Figure 2 legend for expansion of other abbreviation.

The sputum concentration of IL-6 was significantly reduced after simvastatin while reciprocally increased after placebo treatment compared with the baseline (median [IQR], 29.6 pg/mL [9.3-34.2 pg/mL] vs 37.2 pg/mL [18.8-68.3 pg/mL], *P* < .001; 54.0 pg/mL [16.2-79.5 pg/mL] vs 24.4 pg/mL [10.7-52.3 pg/mL], *P* = .02). The suppressive effect of simvastatin therapy was also reflected by a significant reduction in concentrations of sputum CXCL8 compared with placebo treatment (mean ± SD, 447.3 ± 205.1 pg/mL vs 612.7 ± 262.4 pg/mL, *P* < .001; 627.0 ± 225.7 pg/mL vs 602.9 ± 239.9 pg/mL, *P* = .7, respectively). The magnitude of the reduction in IL-6 and CXCL8 concentration was significant in patients with COPD treated with simvastatin compared with subjects treated with placebo (mean difference, −45.3 pg/mL [95% CI, −71.3 to −19.4 pg/mL], *P* = .002; −190.9 pg/mL (95% CI, −324.1 to −57.7), *P* = .007, respectively) (Figs 4A, 4B, Table 3). This suppressive effect was not dependent upon the sequence of treatments in a crossover-designed manner (*P* = .77 for IL-6 and *P* = .87 for CXCL8). Similarly, ICS treatment and smoking status did not influence IL-6 and CXCL8 responses to simvastatin (*P* = .05 and *P* = .09, respectively, for IL-6; *P* = .06 and *P* = .3, respectively, for CXCL8).

### Induced Sputum Cytology

**Figure 5 –:**
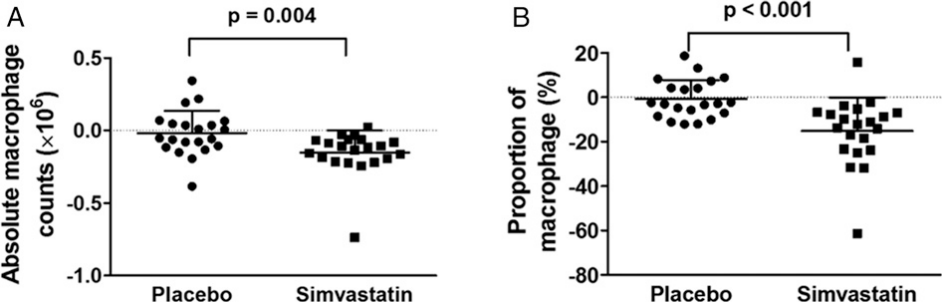
A, Absolute macrophage counts were lower in response to simvastatin treatment to a greater extent than placebo. B, A similar pattern was seen in the proportion of macrophages (% of total cells). The data are expressed as mean (SD).

The total cell counts recovered from sputum were similar after simvastatin and after placebo treatment (Table 3). After 4 weeks, the mean absolute and relative sputum macrophage counts were significantly reduced after simvastatin compared with placebo (mean absolute difference, −0.16 × 10^6^ [95% CI, −0.26 to − 0.06], *P* = .004; −14.1% [95% CI, −21.0 to −7.1], *P* < .001) (Figs 5A, 5B, Table 3). There was a reciprocal increase in the relative proportion of sputum airway epithelial cells (mean proportion difference, 7.6%; 95% CI, 2.3-12.9; *P* = .007) (Table 3), but there was no significant changes in the absolute count of these cells or the counts and proportions of the other sputum cell phenotypes under simvastatin treatment.

### Changes in Clinical Outcomes

Changes in clinical outcomes after simvastatin treatment are listed in Table 4. At 4 weeks, the change in mean CAT score after simvastatin compared with placebo treatment periods was statistically significant (mean difference, −3.2; 95% CI, −6.0 to −0.4; *P* = .02) (Table 4). No statistically significant effect of simvastatin was seen in post-salbutamol FEV_1_, post-salbutamol FVC, or lung resistance at 5 Hz to 20 Hz compared with placebo treatment (Table 4).

**TABLE 4 ]:** The Effect of Simvastatin on Clinical Outcomes

Variables	Placebo	Simvastatin	*P* Value	Treatment Difference, Simvastatin − Placebo (95% CI)	Effects by Order *P* Value
Δ CAT score	0.7 ± 5.5	−2.5 ± 4.1	.02	−3.2 (−6.0 to −0.4)	.5
Δ Post-BD FEV_1_, L	−0.01 ± 0.1	−0.07 ± 0.1	.31	−0.05 (−0.2 to 0.05)	.32
Δ Post-BD FVC, L	−0.04 ± 0.2	−0.01 ± 0.1	.72	0.03 (−0.1 to 0.2)	.82
Δ R5-R20	−0.1 ± 0.9	0.1 ± 0.0	.54	0.2 (−0.48 to 0.88)	.25

Data are presented as mean ± SD. CAT = COPD Assessment Test; R5-R20 = lung resistance at 5-20 Hz. See Table 1 for expansion of other abbreviations.

### Changes in Biochemical Markers

There was no significant change in hsCRP levels between treatment groups (mean difference, −7.7 mg/dL; 95% CI, −19.3 to 4.0; *P* = .18) (Table 5). The biochemical effects of simvastatin therapy were reflected in a significant reduction in concentration of serum low-density lipoprotein cholesterol (mean difference, −47.6 mg/dL; 95% CI, −61.5 to −33.7; *P* < .001), but not high-density lipoprotein cholesterol (mean difference, 1.1 mg/dL; 95% CI, −5.9 to 8.1; *P* = .74) (Table 5). There were no significant differences in mean bilirubin, aspartate aminotransferase, alanine aminotransferase, blood urea nitrogen, creatinine, and creatine phosphokinase levels. No significant adverse events occurred in patients taking simvastatin or in those receiving placebo.

**TABLE 5 ]:** The Effect of Simvastatin on Biologic Markers

Sputum Cells	Placebo (n = 21)	Simvastatin (n = 21)	*P* Value	Treatment Difference, Simvastatin − Placebo (95% CI)	Effects by Order *P* Value
Δ hsCRP, mg/dL	3.6 ± 29.3	−4.0 ± 17.8	.18	−7.7 (−19.3 to 4.0)	.23
Δ LDL, mg/dL	−1.5 ± 24.2	−49.1 ± 23.8	< .001	−47.6 (−61.5 to −33.7)	.51
Δ HDL, mg/dL	−0.2 ± 11.4	0.9 ± 10.0	.74	1.1 (−5.9 to 8.1)	.19
Δ CPK, U/L	5.3 ± 80.1	34.5 ± 45.8	.2	29.2 (−16.0 to 74.4)	.91
Δ Total bilirubin, mg/dL	0.02 ± 0.1	−0.04 ± 0.2	.29	−0.06 (−0.17 to 0.05)	.11
Δ Direct bilirubin, mg/dL	0.01 ± 0.09	−0.01 ± 0.04	.41	−0.02 (−0.07 to 0.03)	.01
Δ AST, U/L	8.2 ± 17.8	2.8 ± 6.4	.17	−5.4 (−13.8 to 2.9)	.88
Δ ALT, U/L	8.9 ± 25.6	0.9 ± 9.1	.16	−8.1 (−19.8 to 3.6)	.76
Δ BUN, mg/dL	0.32 ± 2.7	0.32 ± 3.2	.99	0.01 (−1.8 to 1.8)	.81
Δ Creatinine, mg/dL	0.05 ± 0.09	0.02 ± 0.1	.26	−0.03 (−0.09 to 0.03)	.52

Data are presented as mean ± SD. ALT = alanine aminotransferase; AST = aspartate aminotransferase; BUN = blood urea nitrogen; CPK = creatine phosphokinase; HDL = high-density lipoprotein; hsCRP = high-sensitivity C-reactive protein; LDL = low-density lipoprotein. See Table 1 and 2 legends for expansion of other abbreviations.

## Discussion

This study has demonstrated the ability of simvastatin to suppress Th17 cytokines (IL-17 and IL-22), CXCL8, and IL-6, with concomitant enhancement of IL-10 production and IDO expression and activity in patients with COPD. Surprisingly, simvastatin had no significant effect on neutrophilic airway inflammation, although it did suppress macrophage numbers and improved CAT symptom score.

To our knowledge, no previous study has investigated the antiinflammatory effects of statins in COPD. In particular, the effects on Th17 cytokines, including IL-17 and Il-22, have not been investigated and are unknown in COPD.

Several studies have suggested that COPD may have an autoimmune component that is related to Th17 cells, a subset of CD4^+^ T cells, present in COPD.^21‐23^ Peripheral-blood Th17 cell count was increased in patients with COPD, which was predictive of the severity of airflow limitation.^24^ Th17 cells release IL-17A, IL-17F, and IL-22, all of which have been implicated in the pathogenesis of several inflammatory and autoimmune diseases. IL-17A is a proinflammatory cytokine that regulates airway inflammation and modulates lung and airway structural cells in COPD through the recruitment of inflammatory cells, including neutrophils and lymphocytes. IL-17A acts on airway epithelial cells, smooth muscle cells, and airway fibroblasts to release neutrophil chemoattractants, including CXCL8.^25^ In addition, IL-17 exerts its effect on most parenchymal cells, including macrophages and dendritic cells that express IL-17 receptors, and IL-17-mediated signaling induces the target cells to produce various inflammatory mediators such as tumor necrosis factor-α and IL-6. Studies have shown that the expression of IL-17A and IL-22 was increased in the airways of patients with COPD and that IL-22 can promote airway inflammation by acting in synergy with IL-17A.^26,27^ These data suggest that inhibition of Th17-related cytokines might be a useful strategy for COPD treatment but, so far, no studies with anti-IL-17 or anti-IL-17 receptor antibodies, which are clinically effective in psoriasis and inflammatory bowel disease, have been reported in patients with COPD. Statins have immunomodulatory effects on Th17-mediated human autoimmune response in multiple sclerosis by selectively inhibiting IL-17 transcription and IL-17A, IL-17F, and IL-22 secretion in Th17 cells, and by suppressing Th17-polarizing cytokine release by dendritic cells.^28‐30^ However, little is known about these immunomodulatory effects of statins in patients with COPD. To our knowledge, our results have demonstrated for the first time that simvastatin could markedly suppress secretion of IL-17A and IL-22, which may contribute to decreased IL-6 and CXCL8 production in the airways of patients with COPD. The molecular mechanisms of IL-17A and IL-22 suppression by simvastatin require further investigation. We postulate that there are several mechanisms involving the inhibitory effects of simvastatin on IL-17A and IL-22. Simvastatin can induce SOCS3 expression in dendritic cells that results in suppression of Th17-polarizing cytokine IL-23 release. This effect is associated with geranylgeranylation-dependent inhibition of small GTPases, including RhoA, Rac1, and Rab1, that control IL-23 production.^30^ In addition, simvastatin can directly inhibit IL-17 gene expression in CD4^+^ T cells in a mevalonate-dependent manner.^28^

Treg cells exert their immune-suppressive ability, in part, through secretion of IL-10. A study has shown that the Th17/Treg imbalance, reflected by an increased IL-17A/IL-10 ratio, exists in mice after chronic cigarette-smoke exposure and in patients with stable COPD, and this could play a role in the breakdown of immune self-tolerance in COPD.^11,31,32^ IL-10 transcription and its levels in the airways and lung parenchyma correlated inversely with the severity of COPD and emphysema extent, respectively.^11,33^ In contrast, other studies showed no significant difference in IL-10 levels in BAL from patients with COPD compared with smoker control subjects.^34^ Nevertheless, boosting IL-10 production by simvastatin, as shown in the present study, might restore an impaired immune self-tolerance in patients with COPD. In addition, simvastatin enhanced the expression of the airway immunomodulating enzyme IDO by upregulating its transcription and biologic activity, reflected by the generation of the Tryp metabolite Kyn. Increased IDO activity may lead to Treg cell expansion through the activation of aryl hydrocarbon receptors by Kyn.^35^ Furthermore, accumulation of Kyn resulted in subsequent tolerogenic effects, including increased Treg cell activity.^36^

In a mouse model, the neutrophilic alveolitis associated with acute lung injury is markedly reduced with lovastatin treatment.^37^ We found no significant reduction in the proportion of neutrophils in induced sputum with simvastatin treatment, despite a marked suppression in CXCL8 and IL-17A levels. The absolute neutrophil counts were not significantly different between groups, suggesting that the increased percentage of neutrophils after simvastatin treatment was because of a decrease in percentage of macrophages. In contrast, absolute macrophage counts and the proportion of macrophages were markedly reduced in simvastatin treatment compared with placebo treatment, possibly resulting from the reduction in IL-17A that contributes to macrophage recruitment by activating IL-17A receptors on macrophages.^38^ In keeping with the finding of a reduced sputum macrophage count with simvastatin in COPD, a clinical trial in asthma reported atorvastatin treatment, compared with placebo, was associated with a reduction in the absolute sputum macrophage count.^39^

There are several possible explanations for the apparent lack of clinical effects of simvastatin on airway neutrophilia. The first possibility is that the calculation of sample size in this study had been based on significant reduction in IDO activity but not the proportion of neutrophils and the absolute neutrophil counts. The second possibility is that there was another chemokine driving airway neutrophilia, which could not be suppressed by simvastatin, such as the tripeptide collagen-derived neutrophil chemoattractant N-acetyl proline-glycine-proline (N-ac-PGP), derived from the breakdown of extracellular matrix in the process of alveolar destruction emerging in emphysema. N-ac-PGP plays a role in the airway and parenchymal neutrophilic inflammation that drives COPD progression and exacerbations. N-ac-PGP induced neutrophil chemotaxis and superoxide production through CXCR2 receptors.^40‐42^ In an animal model, the administration of N-ac-PGP caused recruitment of neutrophils into lungs of control mice, but not CXCR2-deficient mice.^40^ In contrast, the blockade of N-ac-PGP with monoclonal antibody suppressed neutrophil responses. N-ac-PGP treatment also caused alveolar enlargement and, therefore, N-ac-PGP activity links degradation of extracellular matrix with neutrophil recruitment in airway inflammation. The considerable concentrations of N-ac-PGP were detectable in the majority of BAL and sputum samples from patients with stable COPD.^40,43^ In addition, cigarette-smoke extract-activated neutrophils can break down collagen to generate N-ac-PGP that can activate neutrophils, leading to the induction of a self-perpetuating cycle of neutrophil infiltration, chronic inflammation, and lung emphysema.^44^ Sputum N-ac-PGP levels were dramatically increased at the time of an exacerbation of COPD, and azithromycin treatment significantly reduced sputum N-ac-PGP levels in patients with COPD.^45^ Suppression of N-ac-PGP may be one of the mechanisms whereby macrolides reduce COPD exacerbations in a clinical trial.^46^ All these data raise the possibility that N-ac-PGP contributes to airway neutrophilia in patients with COPD. In addition, the majority of the patients with COPD in this study were current smokers. It is possible, therefore, that persistent extracellular N-ac-PGP generation induces relentless neutrophilic airway inflammation that is resistant to statin therapy. Other mechanisms driving neutrophilic inflammation in COPD, such as leukotriene B4 or complements, may not be suppressed by a statin, but this requires further studies.

Statins reduce the recruitment of neutrophils and macrophages into the lung.^47,48^ Our results extend the previous findings that the mechanisms by which statins suppress the recruitment of neutrophils and macrophages into the lung are possibly mediated through the inhibition of Th17 cytokines and CXCL8 that could mediate the recruitment. The baseline levels of CXCL8 and IL-6 are associated with the frequency of COPD exacerbations.^49,50^ During COPD exacerbations, CXCL8 and IL-6 levels are significantly elevated in the airways, compared with the stable phase,^51^ suggesting that the development of COPD exacerbations is driven by airway inflammation driven, in turn, by these cytokines. These biologic effects of statins appear to have clinical relevance, as other observational studies reported reduced frequency of COPD exacerbations,^52‐55^ and impeded decline of lung function^56^ in patients taking statins. This was supported by the mechanistic study showing that IL-17 acted as a mediator of IL-1β-induced neutrophilia in influenza-induced COPD exacerbations in mice, and inhibition of IL-17 reduced neutrophil recruitment to the airways both in the initial phase of infection and at the peak of viral replication.^57^ Therefore, biologic effects of statins in COPD demonstrated in the current study may explain the findings from the observational studies.

Our results were in agreement with other observational studies, but differed from the results of a placebo-controlled trial of a statin on COPD exacerbations (STATCOPE [Simvastatin in the Prevention of COPD Exacerbations]). Previous studies showed the beneficial effects of statins on reduced all-cause mortality,^52,58‐60^ including that from acute exacerbation,^52,61^ reduced frequency of COPD exacerbations, especially in patients with COPD who had coexisting cardiovascular disease (CVD),^52‐55,62^ and reduced the decline in lung function.^56^ In contrast, in patients with moderate to severe COPD, the STATCOPE study found no reduction in exacerbations.^63^ A plausible explanation for the discrepancy between the findings of the STATCOPE study and that of the observational studies and our study is the inclusion of a large percentage of patients with COPD with coexisting, overt CVD who were “previous statin users” and “previous statin nonusers,” but would benefit from statin therapy.^64^ These patients are likely to carry a poor prognosis because of one or a combination of the following: undertreated pulmonary inflammation, unrecognized systemic inflammation, or subclinical CVD.^65‐67^ These comorbid phenotypes of COPD are strongly associated with an increased risk of hospitalization with acute exacerbations and greater mortality.^65‐67^ Hence, it is possible that many patients with COPD who have not been prescribed statins in observational studies are undertreated, a hypothesis suggested by STATCOPE investigators to explain this discordance.^63^ Another explanation for the STATCOPE findings is that statin therapy has no effect on reducing acute exacerbations in patients with COPD where coexisting clinical and subclinical CVD has been all but excluded.^64^ The presence of cardiovascular comorbidity underlying acute exacerbations of COPD, in particular heart failure,^68,69^ may intensify the beneficial effect of statins. In addition, the selection of patients with COPD recruited to the STATCOPE study might include a group with much lower risk and who are potentially unresponsive to statin treatment. Any benefit in such a group might be masked.^64^ This was supported by the 23% reduction in low-density lipoprotein levels in the STATCOPE study, with 40 mg of simvastatin, which was less than the expected 36% to 40% reduction normally observed.^64^ Mortality in the placebo arm of the STATCOPE study over 3 years was 6%, only one-half that reported in the Towards a Revolution in COPD Health (TORCH) study over a similar time.^70^ Moreover, the prevalence of comorbid cardiovascular-related diseases in the STATCOPE participants was not reported but is presumably very low or nonexistent.^64^ The exclusion of cardiovascular comorbidity raises questions about the generalization of STATCOPE findings to COPD populations in general, where overt or subclinical CVD may be associated with approximately 75% of all subjects.^64^ Since participants in the STATCOPE study were prescreened through medical record data, it is impossible to estimate what proportion of patients with COPD were excluded because of their cardiovascular profile alone. The exclusion would leave only 20% to 30% “lower risk” patients with COPD eligible for the STATCOPE study.^64^

An alternative explanation for the discrepancy is that the differences among COPD subpopulations under consideration may have masked any beneficial effects of statins in the STATCOPE study. According to the STATCOPE protocol, any patients who met exclusion criteria based on the risk-based eligibility for statin therapy during follow-up could be prescribed simvastatin and continue in the study on an intent-to-treat basis.^63^ The proportion of these patients was not stated in the published STATCOPE study findings. If it was large, a dilutional effect on outcome may have resulted. In addition, patients with COPD in the STATCOPE study were representative of moderate to severe COPD, albeit the use of supplementary oxygen (significantly greater at > 40%) and duration of statin therapy (significantly shorter, with 56% < 2 years) were also substantially different than those in other observational studies.

Despite its novel findings, our study still has potential limitations. Because the present study included only patients with GOLD stage II (moderate) COPD, the results could not necessarily be extended to patients with severe COPD. In addition, because the study had a randomized, cross-over design, prolonged carry-over effects cannot be excluded. However, we attempted to minimize this as much as possible by extending the washout period for 4 weeks. Finally, the number of patients in this study was relatively small and, therefore, a larger number of patients with COPD is required for the future studies to confirm our observations and to study long-term consequences of statin therapy on exacerbations and disease progression.^63^

## ABBREVIATIONS

CAT: COPD Assessment Test
CVD: cardiovascular disease
ELISA: enzyme-linked immunosorbent assay
GOLD: Global Initiative for Chronic Obstructive Lung Disease
hsCRP: high-sensitivity C-reactive protein
ICS: inhaled corticosteroid
IDO: indoleamine 2,3-dioxygenase
IQR: interquartile range
Kyn: kynurenine
LABA: long-acting β_2_-agonist
[M+H]+: protonated species
N-ac-PGP: N-acetyl-proline-glycine-proline
STATCOPE: Simvastatin in the Prevention of COPD Exacerbations
Th: T helper
Treg: T regulatory
Tryp: tryptophan
